# Characterization of MHC class I in a long distance migratory wader, the Icelandic black-tailed godwit

**DOI:** 10.1007/s00251-017-0993-7

**Published:** 2017-05-23

**Authors:** Sara Pardal, Anna Drews, José A. Alves, Jaime A. Ramos, Helena Westerdahl

**Affiliations:** 1 MARE - Marine and Environmental Sciences Centre, Department of Life Sciences, University of Coimbra, 3000-456 Coimbra, Portugal; 2 MEEL - Molecular Ecology and Evolution Laboratory, Lund University, Ecology building, SE-223 62 Lund, Sweden; 3 CESAM - Centre for Environmental and Marine Studies, Department of Biology, University of Aveiro, Campus Universitário de Santiago, 3810-193 Aveiro, Portugal; 4 South Iceland Research Centre, University of Iceland, Fjolheimer, IS-800 Selfoss, Iceland

**Keywords:** Major histocompatibility complex, MHC class I, *Limosa limosa islandica*, Charadriiformes

## Abstract

The major histocompatibility complex (MHC) encodes proteins that are central for antigen presentation and pathogen elimination. MHC class I (MHC-I) genes have attracted a great deal of interest among researchers in ecology and evolution and have been partly characterized in a wide range of bird species. So far, the main focus has been on species within the bird orders Galliformes and Passeriformes, while Charadriiformes remain vastly underrepresented with only two species studied to date. These two Charadriiformes species exhibit striking differences in MHC-I characteristics and MHC-I diversity. We therefore set out to study a third species within Charadriiformes, the Icelandic subspecies of black-tailed godwits (*Limosa limosa islandica*). This subspecies is normally confined to parasite-poor environments, and we hence expected low MHC diversity. MHC-I was partially characterized first using Sanger sequencing and then using high-throughput sequencing (MiSeq) in 84 individuals. We verified 47 nucleotide alleles in open reading frame with classical MHC-I characteristics, and each individual godwit had two to seven putatively classical MHC alleles. However, in contrast to previous MHC-I data within Charadriiformes, we did not find any evidence of alleles with low sequence diversity, believed to represent non-classical MHC genes. The diversity and divergence of the godwits MHC-I genes to a large extent fell between the previous estimates within Charadriiformes. However, the MHC genes of the migratory godwits had few sites subject to positive selection, and one possible explanation could be a low exposure to pathogens.

**Electronic supplementary material:**

The online version of this article (doi:10.1007/s00251-017-0993-7) contains supplementary material, which is available to authorized users.

## Introduction

The major histocompatibility complex (MHC) plays an important role in adaptive immunity (Murphy and Weaver 2017). MHC class I (MHC-I) and class II (MHC-II) genes, encoding MHC-I and MHC-II proteins known to be important for antigen presentation, are highly polymorphic, and this characteristic along with the function in adaptive immunity makes them particularly relevant for studies of molecular evolution. The molecular footprints in MHC-I and MHC-II genes, as well as MHC allele frequencies within populations, are expected to reflect adaptive processes both within and among populations (Hess and Edwards 2002; Sommer 2005). Though MHC-I and MHC-II proteins have a similar overall structure, they operate in slightly different ways by presenting peptides (antigens) mainly from the intracellular (representing e.g., viruses) and extracellular (representing e.g., nematodes and many bacteria) environments respectively (but see crosspresentation, Neefjes et al. 2011; Murphy and Weaver 2017). MHC-I proteins are found on all nucleated cells, and if the presented peptide is the product of a foreign or transformed antigen, it is recognized as foreign by T cells which will kill the infected/transformed cell (Murphy and Weaver 2017). Antigen presentation by MHC proteins is the key initial step for triggering an adaptive immune response, and the peptide-binding residues (PBR) of the MHC proteins are therefore particularly interesting when studying evolutionary processes, such as host-pathogen interactions at the molecular level.

The first studies of MHC characterization in avian species were conducted in the 1950s using the domestic chicken (*Gallus gallus domesticus*) as a model species. Domestic chickens have two classical MHC-I genes, and birds in general were, for a long time, thought to possess few MHC genes (Kaufman et al. 1999). Classical MHC genes encode cell-surface molecules that have high polymorphism, wide tissue distribution, and present peptides to T cells, whereas non-classical class I genes lack one or more of these properties (Shawar et al. 1994; Rodgers and Cook 2005). Subsequent studies on MHC-I gene expression of additional Galloanserae species, such as the Japanese quail (*Coturnix japonica*) and the mallard (*Anas platyrhynchos*), found just one highly expressed classical MHC-I gene copy, further supporting the belief that birds in general have, or express, a limited number of MHC-I genes (Shiina et al. 2006; Moon et al. 2005). However, partial characterization of MHC-I in additional bird orders, Passeriformes (songbirds) in particular, have proven the existence of large numbers of MHC-I gene copies at the genomic level (Westerdahl et al. 1999; Sepil et al. 2012; Karlsson and Westerdahl 2013; O’Connor et al. 2016; but see Balakrishnan et al. 2010), though the number of expressed MHC-I genes is yet to be thoroughly investigated (Westerdahl et al. 1999; Karlsson and Westerdahl 2013; Drews et al. 2017).

When high-throughput sequencing (HTS) became feasible for large scale MHC genotyping in non-model organisms, it boosted the number of bird species for which there are good estimates of the total number of MHC alleles per individual (Zagalska-Neubauer et al. 2010; Sepil et al. 2012; Karlsson and Westerdahl 2013). MHC diversity (including e.g., number of segregating sites, allelic divergence, and nucleotide diversity) has primarily been studied within species on a micro-evolutionary scale, but more recently also on the macro-evolutionary level across species (O’Connor et al. 2016), and also including the MHC genomic organization (Wang et al. 2014; Chen et al. 2015). Nonetheless, these HTS studies on MHC diversity have so far mostly been restricted to passerines, while there is a lack of knowledge in other bird orders, hence, limiting the potential for broader comparative analyses. We therefore set out to firstly partly characterize MHC-I and secondly genotype MHC-I diversity in a species from the bird order Charadriiformes.

Species within Charadriiformes are particularly interesting from a host-pathogen perspective, since this group includes long-distance migrants with a wide range of migratory strategies resulting in striking differences in pathogen exposure (Piersma 1997, 2003; Delany et al. 2009; Clark et al. 2015). MHC-I genes have previously been partly characterized in two distantly related Charadriiformes species, the red knot (*Calidris canutus*; hereafter knot), and the red-billed gull (*Larus scopulinus*; hereafter gull) (Cloutier et al. 2011; Buehler et al. 2013). Interestingly, the MHC-I diversity and MHC organization in knots and gulls differs considerably. Our study species, the black-tailed godwits (*Limosa limosa*), specifically the Icelandic subspecies (*Limosa limosa islandica*; hereafter godwits), is restricted to relatively pathogen-free areas (Piersma 1997, 2003; Dobson et al. 2008; Davidson et al. 2011), and we therefore expect low MHC-I diversity. In the present study, we (1) partly characterize MHC-I in godwits using Sanger sequencing on long transcripts, (2) genotype MHC-I alleles in a breeding population of godwits using HTS (Illumina MiSeq), (3) investigate genetic diversity (measured as number of segregating sites and nucleotide diversity), evolutionary divergence, and selection in godwit MHC-I alleles, and (4) finally compare the MHC-I genetic organization and MHC diversity in three species from the order Charadriiformes, godwits, knots, and gulls.

## Material and methods

### Study subspecies and fieldwork

The godwit is a migratory shorebird distributed over large discontinuous breeding and wintering ranges, and within the western Palearctic, only the nominate (*Limosa limosa limosa*) and Icelandic subspecies occur (Delany et al. 2009). The Icelandic subspecies breed predominantly on Iceland, and outside the breeding season, the birds move along the Atlantic coast, often in brackish habitats such as sheltered estuaries, lagoons, and large intertidal mudflats, to winter in temperate countries (mostly Iberia) (Gill et al. 2007; Delany et al. 2009; Alves et al. 2010; Alves et al. 2013). We captured incubating adults during the breeding season (May to July of 2011, 2012, and 2013) on the southwestern part of Iceland (64° 1′ 46.14″ N, 20° 59′ 6.04″ W) with the use of nest-traps. Captured birds were blood sampled from the brachial vein (ca. 90 μl), ringed, and released without deliberate harm. Blood for genomic DNA (gDNA) was kept in 96% ethanol, while for RNA, blood was transferred to a NUNC tube filled with 500 μl RNAlater (ThermoFisher Scientific/Ambion, Waltham, USA). DNA and RNA samples were kept at −20 °C, during fieldwork and then stored at −80 °C until laboratory analysis. In total, this study is based on 84 samples of genomic DNA collected in 2011–2013, and one RNA sample collected in 2013.

### Extraction, gDNA, and cDNA preparation

Total genomic DNA was extracted by an adapted ammonium acetate protocol (Richardson et al. 2001). RNA extraction and purification was done using a combination of the TRIzol LS manufacturer protocol (Life Technologies, Carlsbad, CA, USA) and the RNeasy Mini kit manufacturer protocol (QIAGEN, Hilden, Germany). To reverse transcribe the messenger RNA (mRNA) to complementary cDNA, we used the RETROscript Kit according to the manufacturer’s instructions (ThermoFisher Scientific/Ambion, Waltham, USA). See online resource Material and Methods for detailed information.

### Long MHC-I transcripts: primer design, molecular cloning, and Sanger sequencing

**Fig. 1:**
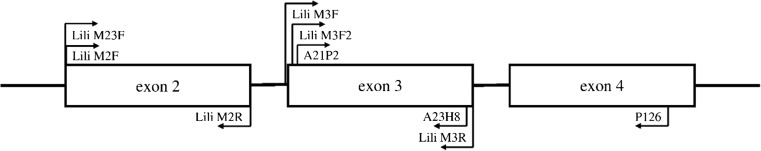
Schematic representation of an MHC-I gene (intron 1, exon 2, intron 2, exon 3, intron 3, exon 4, and intron 4) where *arrows* indicates the position of the nine primers used in the present study. Forward primers are represented *above* the exons, while the reverse primers are shown *below*. Primers A21P2 and P126 come from Strandh et al. (2011), and the primer combination LiliM3F and LiliM3R was used for Illumina MiSeq sequencing

Primer pairs for amplification of MHC-I in the godwits were either newly designed or from a previous study by Strandh et al. (2011) (Fig. 1; online resource Table S1). A first set of new primers that amplified approximately 736 bp, covering partial exon 2 to partial exon 4, was designed based on an alignment consisting of MHC class I sequences from domestic chickens (NM001030675.1; HQ141386.1), Japanese quail (AB005528.1), mallard ducks (AB115239.1), great reed warblers (*Acrocephalus arundinaceus*, AJ005503.1), red-billed gulls (HM008714.1; HM008715.1; HM008713.1; HM008716.1), blue petrels (*Halobaena caerulea*, JF276881.1; JF276884.1), red knots (KC205119.1; KC205120.1, KC205121.1; KC205116.1; KC205117.1; KC205107.1; KC205113.1), and Florida sandhill cranes (*Grus canadensis pratensis*, AF033106.1) downloaded from the GenBank database (Benson et al. 2013). By adding successful godwit cDNA and gDNA sequences (i.e., exon 2, exon 3, and exon 4 sequences) to the previous alignment, new sets of primers could be designed for amplifying exons 2 and 3. The combined sequence data from many primer pairs gave satisfactory coverage of the variable exons 2 and 3, the exons that encode the peptide binding region (519 bp out of 540 bp in this region was successfully sequenced). All MHC fragments were amplified using 10 ng of cDNA or 25 ng gDNA as template with standard PCR protocols, AmpliTaq polymerase kits (Applied Biosystems, New Jersey, USA), and the annealing temperatures are specified in the online resource Table S1. The PCR products were visualized on 2% agarose gels using electrophoresis. PCR products of the correct lengths were cloned using the TOPO TA Cloning Kit with the pCR2.1 TOPO vector and One Shot chemically competent cells, following the manufacturer’s instructions (Invitrogen, Paisley, UK). Positive bacteria colonies (white) were put in 150 μl of double-distilled water and heated to 95 °C for 3 min in order to lyse the cells. Afterwards, PCR amplification was performed using 1–2 μl of lysed bacteria as template, using the forward primer M13F and the reverse primer M13R, these primers are designed to amplify inserts in the cloning vector (Invitrogen, Paisley, UK). To determine which bacterial colonies that had inserts of correct length, the PCR products were again run on 2% agarose gels. Successful PCR samples were precipitated using 11 μl 8.0 M ammonium acetate and 37.5 μl 95% ethanol. Finally, the precipitated and purified PCR product was used as template for Sanger sequencing using the BigDye terminator kit v.3.1 (Applied Biosystems, New Jersey, USA). Sequences were obtained from an ABI PRISM 3130 genetic analyzer (Applied Biosystems, New Jersey, USA), and the results were then edited in BioEdit 7.2.5 Sequence Alignment Editor (Hall 1999) and Geneious version 8.1.7 (http://www.geneious.com; Kearse et al. 2012). Altogether, 85 cDNA and 15 gDNA high quality DNA sequences were retrieved from a single godwit individual using nine different primers in seven different combinations.

### Illumina MiSeq sequencing of MHC-I exon 3

The new godwit MHC-I exon 3 primers, Lili M3F (5′-TCGYGTTCCAGGGGCTCACA-3′) and Lili M3R (5′-GGCYGTGCTGGAGAGGAAA-3′), were designed based on the abovementioned alignment of exons 2 and 3 and used for Illumina MiSeq MHC-I genotyping. The primer pair satisfactorily amplified 244 and 247 bp long fragments in all 84 godwit individuals. Each 25 μl amplicon PCR reaction contained 12.5 μl 2X Phusion High-Fidelity PCR Master Mix (ThermoFisher Scientific, Waltham, USA), 0.5 μM of each primer, and 25 ng of gDNA template. The two-step PCR profile was set to 25 cycles 98 °C (10 s) and 72 °C (15 s), ending with 72 °C for 10 min. PCR products were checked on a 2% agarose gel and then cleaned with Agencourt AMPure XP-PCR Purification kit (Beckman Coulter, Indianapolis, USA), following the manufacturer’s instructions. The PCR clean-up was done by adding 20 μl AMPure XP beads to each reaction, washing with 75% ethanol, and adding 43 μl of double-distilled water for elution.

To allow recovery of individual amplicons after demultiplexing, unique combinations of forward and reverse Illumina indexes were added to each sample by using Nextera XT v2 Index Kit (Illumina Inc., San Diego, CA, USA). Twenty-five microliters PCR reaction volume were prepared containing 12.5 μl 2X Phusion High-Fidelity PCR Master Mix (ThermoFisher Scientific, Waltham, USA), 3 μl of each index primers, and 2 μl of cleaned PCR amplicon product. The PCR profile was set to 8 cycles at 98 °C (10 s), 55 °C (30 s), and 72 °C (15 s), ending with 72 °C for 5 min. The PCR products were checked on a 2% agarose gel and cleaned with Agencourt AMPure XP-PCR Purification kit (Beckman Coulter, Indianapolis, USA). Cleaning was done as mentioned above, except for the addition of 23 μl AMPure XP beads to each sample and 38 μl of double-distilled water for elution. Cleaned PCR products were then checked on a 2% agarose gel, and the concentration was measured with Quant-iT PicoGreen dsDNA Assay Kit (ThermoFisher Scientific/Invitrogen, Waltham, USA) modified for a 96-well plate. Equimolar quantities of every sample were pooled, and these pools were then quantified with Qubit (ThermoFisher Scientific, Waltham, USA) and run on a Bioanalyzer DNA 2100 chip for quality and size validation. Lastly, equimolar quantities of all pools were taken together to create a final 4 nM library that was sent for 300 bp paired-end Illumina MiSeq sequencing (Illumina Inc., San Diego, CA, USA) at the DNA sequencing facility Department of Biology, Faculty of Science, Lund University.

### Filtering Illumina MiSeq data

For bioinformatic post-processing of HTS data, we used the Amplicon Sequencing Analysis Tools (AmpliSAT) (web server http://evobiolab.biol.amu.edu.pl/amplisat/; Sebastian et al. 2015) for demultiplexing, clustering, and filtering. Clustering and filtering to remove artefacts from our dataset was performed in four steps having in mind key assumptions based upon previously described methods (Galan et al. 2010; Lighten et al. 2014; Stutz and Bolnick 2014); for more details, see online resource Material and Methods. Step (1), to make sure that each amplicon had a reliable read depth for allele characterization, a linear plot of amplicon read depths was produced. This allowed us to detect and remove poor quality amplicons (i.e., with low read depth) that could introduce bias into the analysis. The minimum required read depth was set to 4000 reads per amplicon. Step (2), in order to reassign reads arising from artefacts to the parental sequences from which they arose, we used the clustering function in AmpliSAT (Sebastian et al. 2015). Artefact sequences were merged with the dominant sequences (presumed parent sequences) when they differed by 1–2 bp and had ≤25% of read depth compared to the dominant sequences. Sequences that differed by 1–2 bp from the dominant sequences but with higher read depth than 25% were classified as “subdominants” and formed a new cluster. Step (3), for the establishment of a suitable per amplicon frequency, since there is no information regarding the number of MHC-I loci for godwits, the threshold was determined by the best match between technical duplicates (*n* = 6 samples) in a similar fashion to Karlsson and Westerdahl (2013) and O’Connor et al. (2016). Best matches were obtained with a per amplicon frequency of 3.4%, and any sequences occurring below this value were considered to be artefacts and removed. Finally, during step (4) chimeric sequences were identified by using the chimera checking function within AmpliSAT (in our data set the highest frequency of chimeras was 0.6%, hence, much lower than the threshold set to 3.4%).

## Data analysis

### Validation and identification of putatively functional alleles

MHC-I alleles from cloning were considered verified if they were found in two independent PCR reactions and/or when sequences matched those found by Illumina MiSeq for the same individual. All MHC-I alleles from Illumina MiSeq that remained in the dataset after filtering were considered verified. All verified alleles from cloning and Illumina sequencing were blasted in GenBank to confirm that they were MHC-I alleles and to check if they had been described previously in any other avian species. Unique alleles were uploaded to the GenBank database and given species-specific names, following the Klein et al. (1990) nomenclature for naming MHC alleles (*Mhc*Lili-UA*xx). After manual alignment of alleles in BioEdit 7.2.5 Sequence Alignment Editor, the DNA sequences translated into amino acid sequences, all in open reading frame.

### Analysis of allelic diversity, recombination, and rates of positive selection in godwits

The MHC-I exon 3 sequences were analysed in MEGA 7.0.14 (Kumar et al. 2016). The analyses included estimation of the number of segregating amino acid sites (*S*_aa_), average nucleotide diversity (π), evolutionary divergence (number of differences per site from averaging over all sequence pairs) for nucleotide sequences (*d*_nt_), and amino acid sequences (*d*_aa_). The divergence tests were estimated using the Kimura 2-parameter (K2P) model (Kimura 1980) with a gamma distribution (*α* = 1) or with a p-distance model with uniform rates, both these tests were run with 1000 bootstrap repeats. All the analyses were performed by including three different sets of sequences, only alleles without a 3 bp deletion (247 bp long, hereafter called “long alleles”, *n* = 38), alleles with a 3 bp deletion (244 bp long, hereafter called “short alleles”, *n* = 9), or for all alleles simultaneously (i.e., long and short alleles combined, *n* = 47) (corresponding *n* values for alleles based on amino acid sequences were 31, 9, and 40). To get an overview of the allele frequencies in the godwit population, histograms of mean allele frequencies were created in SigmaPlot (Systat Software, San Jose, CA) (online resource Fig. S1).

**Fig. 2:**
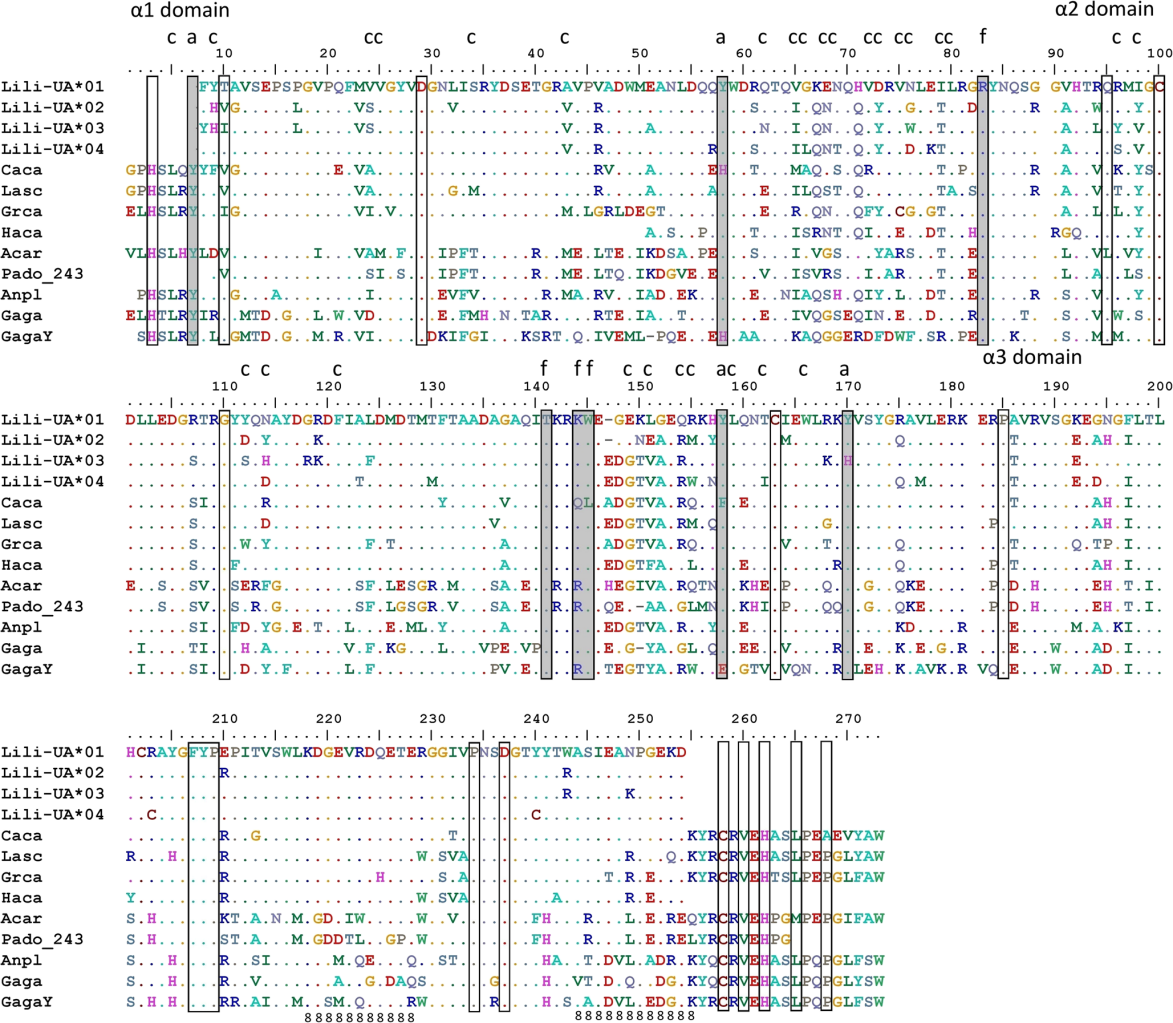
Amino acid alignment of Icelandic black-tailed godwit (*Limosa limosa islandica*; Lili) MHC-I alleles covering the *α*1, *α*2, and *α*3 domains (the first residue of the alignment is the first residue in *α*1). Identity to Lili-UA*01 is indicated by *dots*, a *blank space* determines the end of an exon, and *dashes* indicate true gaps. *Transparent boxes* indicate inter- and intra-domain contact residues, *light grey boxes* indicate peptide main chain sites, *c* represents non-main chain peptide contacts, *a* and *f* indicate the A and F pocket, and *8* indicates the CD8 binding sites (Bjorkman et al. 1987; Saper et al. 1991; Grossberger and Parham 1992; Kaufman et al. 1994; Wallny et al. 2006). NCBI sequence sources are Caca (red knot, *Calidris canutus*, KC205116), Lasc (red-billed gull, *Larus scopulinus*, HM025963), Grca (florida sandhill crane, *Grus canadensis pratensis*, AF033106), Haca (blue petrels, *Halobaena caerulea*, JF276884), Acar (great reed warblers, *Acrocephalus arundinaceus*, AJ005503), Pado (house sparrow, *Passer domesticus* with 3 bp deletion, Pado_243, Karlsson and Westerdahl 2013), Anpl (mallard ducks, *Anas platyrhynchos*, GU245878), Gaga (chickens, *Gallus gallus domesticus* BF2, HQ141386), and GagaY (chickens, *G. g. domesticus* YF6, XM_003643736)

**Fig. 3:**
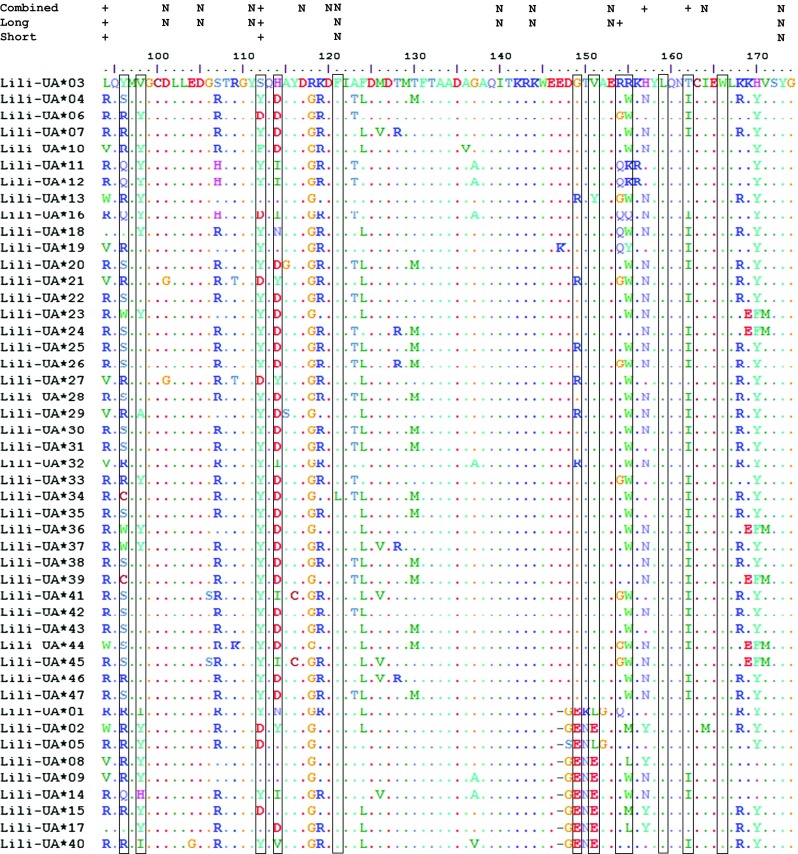
Alignment of 47 Icelandic black-tailed godwit MHC-I alleles covering 244–247 bp of the *α*2 domain (exon 3) sequenced with Illumina MiSeq. Identity to Lili-UA*03 is indicated by *dots*, and *dashes* indicate true gaps. Alleles Lili-UA*01, 02, 05, 08, 09, 14, 15, 17, and 40 have a 3 bp deletion at position 147. Alleles Lili-UA*04, 28, 30, 47 encode for the same amino acid, as does Lili-UA*7, 46; Lili-UA*11, 12; Lili-UA*22, 42, and Lili-UA*35, 43. The *boxes* indicate the peptide-binding residues (PBR) inferred from Wallny et al. (2006), excluding peptide main-chain sites. Residues under positive selection (+) and negative selection (N) were calculated using the SLAC, FEL, and REL methods (www.datamonkey.org; Pond et al. 2006; Delport et al. 2010)

Average rates of synonymous (*d*_S_) and nonsynonymous (*d*_N_) substitutions in the PBR and non-PBR were estimated in MEGA 7.0.14 (Kumar et al. 2016), and in the fixed-site model to infer positive selection (dN/dS ratio), 12 PBR in exon 3 were included (residue 96, 98, 112, 114, 121, 149, 151, 154, 155, 159, 162, and 166, see Fig. 3 for details). Again, the analyses were done either for all alleles simultaneously (long and short nucleotide alleles; *n* = 44, the *n* value dropped from 47 to 44 since 2 bp in the beginning of the sequence and 2 bp in the end of the sequence were incomplete codons), for long alleles only (*n* = 35; the *n* value dropped from 38 to 35 due to incomplete codons), or for short alleles only (*n* = 9). The PBR were inferred in the long transcripts in accordance with the previously documented PBR in human and chicken MHC-I (Wallny et al. 2006) which overlap with described patterns of positive selection in other bird species (e.g., Alcaide et al. 2009; Cloutier et al. 2011; Buehler et al. 2013). The *α*1 (exon 2) PBR were the following 5, 7, 9, 24, 25, 34, 43, 58, 62, 65, 66, 68, 69, 72, 73, 75, 76, 79, 80, and 83, and in *α*2 (exon 3), they were 96, 98, 112, 114, 121, 141, 144, 145, 149, 151, 154, 155, 158, 159, 162, 166, and 170 (see Fig. 2 for details).

Before testing for evidence of positive selection with maximum likelihood (ML) methods, we explored the presence of recombination breakpoints using the genetic algorithm for recombination detection (GARD), available through the Datamonkey webserver (www.datamonkey.org; Pond et al. 2006; Delport et al. 2010). These analyses were again done using the three different sets of nucleotide alleles, short and long alleles combined (*n* = 40 randomly selected nucleotide sequences out of 44; the *n* value dropped from 47 to 44 due to incomplete codons, and 40 is the maximum allowed number of sequences for REL analysis), for long alleles only (*n* = 35; the *n* value dropped from 38 to 35 due to incomplete codons), or for short alleles (*n* = 9). Inferred GARD trees were then used to perform analysis of positive selection with ML methods. Priori fixed-site model do not always best describe positively selected sites, since selection (positive or negative) might act outside the inferred PBR, particularly so in non-model species (Yang and Swanson 2002). Random sites models, like ML, can thus be better at identifying positive selected sites, since it describes the overall variation among sites (Furlong and Yang 2008). Evidence for positive selection was therefore inferred from combining the results of the following ML methods: single-likelihood ancestor counting (SLAC) at *P* = 0.1, fixed effects likelihood (FEL) at *P* = 0.1, and random effects likelihood (REL) with BF > 50 (online resource Table S2), all performed on the Datamonkey webserver (Pond et al. 2006; Delport et al. 2010).

### Diversity, selection, and phylogenetic distances of MHC-I alleles from three Charadriiformes species

The MHC diversity and phylogenetic distances of MHC-I exon 3 alleles from putatively classical MHC-I alleles were compared between the three Charadriiformes species, godwits, knots, and gulls. There were 21 different putatively classical MHC-I alleles in GenBank from gulls, and we therefore decided to use 21 sequences from each Charadriiformes species; gulls (Lasc-UAA*01; Lasc-UAA*03-16; Lasc-UBA*02-06; Lasc-UBA*08 (Cloutier et al. 2011)), 21 random sequences from knots (Caca-UA*01-02; Caca-UA*04; Caca-UA*06-09; Caca-UA*11-15; Caca-UA*17-25 (Buehler et al. 2013)), and 21 random sequences from godwits. We then calculated number of segregating nucleotide (*S*_nt_) and amino acid sites (*S*_aa_), *d*_N_, and *d*_S_ in the three Charadriiformes species as described above.

Phylogenetic networks allow the representation of alternative phylogenetic histories, and besides the assumption of mutation and speciation events, networks also takes into account gene loss, duplication, and recombination (Bryant and Moulton 2004) processes which are known to affect MHC gene evolution (Nei et al. 1997; Hess et al. 2002; Spurgin et al. 2011). Charadriiformes species-specific neighbour-networks on exon 3 were built using SplitsTree v.4.14.4 (Huson and Bryant 2006) based on the substitution model K2P. The best-fitting nucleotide substitution model was determined with the JModel test 2.1.10 (Posada 2008). In these networks, we included both putatively classical and non-classical MHC-I alleles. We downloaded a representative subsample of 32 exon 3 MHC-I alleles from knots (Caca-UA*01-02; Caca-UA*04; Caca-UA*06-09; Caca-UA*11-15; Caca-UA*17-36; Buehler et al. 2013) and gulls (Lasc-UAA*01; Lasc-UAA*03-16; Lasc-UBA*02-06; Lasc-UBA*08; Lasc-UCA*01-04; Lasc-UDA*01–04; Lasc-UDA*06; 08 and 09; Cloutier et al. 2011) from the GenBank database. We decided to include 47 exon 3 MHC-I alleles in the godwit network, which covered either the entire exon 3 (i.e., from Sanger sequences, 276 bp; Lili-UA*01-04) or the main part of exon 3 (i.e., from Illumina MiSeq, 244 and 247 bp; Lili-UA*05-47). For godwits, the model was run with a transition/transversion (R) rate of 1.9421, probability of invariable sites of 0.4470 (p-inv) and a gamma distribution shape parameter (*α*) of 0.4250. For knots, the model was run with R rate of 1.3753, p-inv of 0.3220, and an *α* of 0.5000, and for gulls, we used an R rate of 1.4271, p-inv of 0.5500, and an *α* of 0.5750. In all networks, 1000 bootstrap repeats were used.

The phylogenetic relationships of MHC-I alleles (sequences covering exon 2, exon 3, and exon 4) within Charadriiformes (godwits, knots, and gulls) was reconstructed using ML trees in MEGA 7.0.14 (Kumar et al. 2016). Selection of the best substitution models was determined using BIC and AICs in MEGA, and phylogenetic analyses were performed simultaneously for exon 2–3 and separately for exon 4, using a chicken MHC-I sequence as an outgroup. Nucleotide-based trees were built using T92 models (Tamura 1992), while amino acid trees were inferred by JTT models (Jones et al. 1992), both using 1000 bootstrap replication.

## Results

### Black-tailed godwit MHC-I, the *α*1, *α*2, and *α*3 domains

Sanger sequencing using nine different primers on both gDNA and cDNA from a single godwit individual resulted in 100 confirmed MHC-I sequences of different lengths (Fig. 1; 174–741 bp; online resource Table S3), identifying a total of seven different alleles. From these seven alleles, we obtained transcripts with longer coverage (736–738 bp) of four distinct MHC-I alleles: Lili-UA*01, Lili-UA*02, Lili-UA*03 and Lili-UA*04 (GenBank accession numbers: KY351552-KY351555). These transcripts cover the major part of exon 2–4: partial *α*1 (82 out of 88 amino acids), entire *α*2 (92 amino acids), and partial *α*3 (72 out of 91 amino acids). The godwit MHC-I amino acid sequences were easily aligned to MHC sequences from other birds (Fig. 2). Lili-UA*01 and Lili-UA*02 have a 3 bp deletion at nucleotide positions 415–417 in the *α*2 domain (amino acid position 147 in the alignment), while Lili-UA*03 and Lili-UA*04 contain no deletions. Classical MHC-I amino acid sequences have eight highly conserved sites “YYRTKWYY” (found in non-mammalian vertebrates), so called peptide main-chain sites, and in the godwit MHC-I transcripts, seven of these sites were covered. There was only one deviation from the consensus “YRTKWYY” in the godwit transcripts, the allele Lili-UA*03 had a substitution from tyrosine (Y) to histidine (H) at amino acid position 170 in the alignment. Inter- and intra-domain contact residues are also highly conserved across vertebrates, e.g., the cysteine (C) residues responsible for the disulphide bridge formation (Grossberger and Parham 1992; Kaufman et al. 1994). In the godwit transcripts, 12 out of the 18 inter- and intra-domain contact residues were covered, and out of these, 11 residues remained unaltered, while one position (amino acid position 10 in the alignment) was highly variable (Fig. 2). The CD8 binding region (amino acid positions 218–228 and 244–255 in the alignment) is known to be highly variable between species but conserved within species (Salter et al. 1989; Kaufman et al. 1994). The godwit MHC-I sequences were conserved here, except for allele Lili-UA*03 that had a lysine (K) instead of asparagine (N) at position 249. Gene expression was studied in this single godwit individual, and in addition to the four long transcripts mentioned above, one additional allele was identified in cDNA, indicating that at least five alleles (Lili-UA*01-05) are transcribed (found in RNA) out of the seven (Lili-UA*01–07) found in gDNA.

### Exon 3 sequences (*α*2 domain) from Illumina MiSeq

The average read depth per amplicon, i.e., the number of MiSeq Illumina sequence reads per sample after filtering the MiSeq data, was 11,322 ± 335 (x^_^ ± SE). The sequences from 90 amplicons (*n* = 84 individuals; *n* = 6 duplicates; all from gDNA) were either 244 or 247 bp, and there was 100% match in the MHC genotypes of each duplicate pair. These MiSeq exon 3 sequences covered 81 out of the 92 amino acids in the *α*2 domain. Forty-seven MHC-I alleles were verified (GenBank accession numbers: KY351556-KY351598); 38 alleles had no deletions and are referred to as “long alleles,” whereas nine alleles had a 3 bp deletion at amino acid position 147, and these alleles are referred to as “short alleles”. We decided to keep all alleles that fulfilled all the criteria set for the genotyping, and we therefore kept seven alleles that were only found in a single individual, these alleles had a per amplicon frequency of 7–16% which is much higher than the threshold of artefacts set to 3.4%.

In the individual that was genotyped with both Sanger sequencing and Illumina MiSeq, six out of seven MHC-I alleles were found with both techniques (Lili-UA*02 to UA*07), but allele Lili-UA*01 was not found using MiSeq. The missing allele Lili-UA*01 was found in a very low read depth in the MiSeq amplicon data and was therefore deleted during the filtering process. This is likely to be due to amplification failure in this individual, though the allele Lili-UA*01 was successfully amplified in three other individuals.

The 47 MHC-I alleles from Illumina MiSeq translated into 40 unique amino acid sequences, and more than half (10 out of 18) of the segregating sites were found within the peptide binging region (Fig. 3). Functional characteristics of the inter- and intra-domain contact residues were highly conserved across all alleles, with the exception of tyrosine (Y) at position 170 that was replaced by histidine (H) in four alleles (UA*03, UA*08, UA*09, and UA*14) and by phenylalanine (F) in six alleles (UA*23-24, UA*36, UA*39, and UA*44-45), a pattern seen also in the long transcripts (Fig. 2). Motifs that were unique for the short alleles and long alleles are, for example, “GENE” (at amino acid positions 148–151) and “EDGTV” (at amino acid positions 147–151), respectively (Fig. 3).

### Number of exon 3 alleles per individual and allele frequencies

There were two to seven alleles in total per individual (x^_^ ± SD: 4.92 ± 1.09), and assuming heterozygosity, godwits have between one and four MHC-I loci (online resource Table S4). Among the 84 genotyped individuals, all individuals had long alleles (1–6 per individual; x^_^ ± SD: 3.74 ± 1.11), 18 individuals had only long alleles (2–6 per individual; x^_^ ± SD: 4.39 ± 0.92), and 66 individuals had at least one short allele (1–3 per individual; x^_^ ± SD: 1.50 ± 0.64). As mentioned above, 38 alleles were long and nine were short, and the allele frequency distribution in our study population indicated that long alleles seem to range from common to rare, while short alleles tended to be overall more common and have a more even distribution (online resource Fig. S1).

### Diversity, inference of positive selection, and recombination in exon 3 alleles

There were no striking differences in overall diversity between long and short alleles, though short alleles had slightly higher nucleotide diversity (π) and divergence (nucleotide and amino acid distances) (Table 1). The fixed-site model analysis showed no significant support for positive selection acting on the PBR of exon 3 in any of the three comparisons, though note that the *d*_N_/*d*_S_ ratio was twice as high in the PBR relative to the non-PBR for the long alleles and that the synonymous substitution (*d*_S_) value is twice as high for the short alleles compared with the long alleles (Table 1). This absence of evidence for positive selection in the PBR of the godwit MHC alleles using the fixed-site model is probably due to high rates of dS, particularly in the case of the short alleles.

**Table 1:** Measures of MHC-I diversity (segregating amino acid sites (*S*_aa_) and average nucleotide diversity (π)) and evolutionary divergence (for nucleotide sequences (*d*_nt_) and amino acid sequences (*d*_aa_)) of exon 3 alleles in the Icelandic black-tailed godwit (*Limosa limosa islandica*)*.* These analyses were carried out for all alleles simultaneously (i.e., long and short nucleotide alleles; *n* = 47), for long alleles only (nucleotide alleles, *n* = 38), and for short alleles only (*n* = 9) (corresponding *n* values for amino acid sequences were 40, 31, and 9, respectively). Estimates of non-synonymous (*d*_N_) and synonymous (*d*_S_) substitution rates, plus the *d*_N_/*d*_S_ ratio, were calculated separately for the peptide-binding residues (PBR) and non-PBR (the PBR here were defined as 12 sites in exon 3, see Fig. 3 for details), for all alleles simultaneously (*n* = 44), for long alleles only (*n* = 35), and for short alleles only (*n* = 9)

	*d*_N_ ± SE	*d*_S_ ± SE	*d*_N_/*d*_S_	*S*_aa_	π	*d*_nt_ ± SE	*d*_aa_ ± SE
All							
PBR	0.322 ± 0.104	0.310 ± 0.160	1.039	10	0.255	0.560 ± 0.285	0.422 ± 0.075
Non-PBR	0.046 ± 0.010	0.082 ± 0.025	0.561	27	0.041	0.044 ± 0.016	0.103 ± 0.020
Long							
PBR	0.239 ± 0.086	0.209 ± 0.148	1.144	10	0.220	0.438 ± 0.245	0.335 ± 0.067
Non-PBR	0.040 ± 0.009	0.059 ± 0.022	0.678	23	0.039	0.043 ± 0.016	0.091 ± 0.019
Short							
PBR	0.302 ± 0.107	0.394 ± 0.179	0.766	8	0.375	0.505 ± 0.310	0.375 ± 0.093
Non-PBR	0.041 ± 0.012	0.078 ± 0.027	0.526	14	0.089	0.041 ± 0.019	0.089 ± 0.023

Selection estimation by ML methods, on long and short alleles combined, identified four sites subject to positive selection in exon 3 (positions 94 (non-PBR), 112 (PBR), 157 (non-PBR), and 162 (PBR) in Fig. 3), and two of these fall outside the PBR inferred by Wallny et al. (2006). When this analysis was run on the 35 long alleles only, it identified three positively selected sites (positions 94 (non-PBR), 112 (PBR), and 154 (PBR)). When running the ML analysis on the nine short alleles, two previously identified positive selected sites were found (position 94 (non-PBR) and 112 (PBR)). In the latter analysis, the few sites subjected to positive selection can partly be explained by the low number of sequences included in the analysis.

Recombination was investigated separately for the long and short alleles. There were no recombination points in the long alleles, but GARD predicted one recombination point at amino acid position 121 in the nine short alleles.

### MHC-I diversity and phylogenetic relationships among three Charadriiformes species

When comparing the godwit exon 3 diversity with that of two other Charadriiformes species, the knot and the gull, the godwit measures of diversity for the non-PBR fell in between those of the two other species (Table 2). However, the *d*_N_/*d*_S_ ratio in the PBR was lower for godwits than for either gulls or knots, probably explained by the high dS in godwits. Moreover, the number of segregating sites in the PBR was highest in godwits, while the nucleotide diversity fell in between that of gulls and knots.

**Table 2:** Measures of MHC-I diversity (segregating nucleotide (*S*_nt_) and amino acid sites (*S*_aa_) and average nucleotide diversity (π)) of exon 3 alleles in Icelandic black-tailed godwits (*Limosa limosa islandica*), red-billed gulls (*Larus scopulinus*), and red knots (*Calidris canutus*)*.* All analyses were carried out on 21 putatively classical alleles per species. Estimates of non-synonymous (*d*_N_) and synonymous (*d*_S_) substitution rates, plus the *d*_N_/*d*_S_ ratio, were calculated separately for the peptide-binding residues (PBR) and non-PBR (the PBR here were defined as 12 sites in exon 3, see Fig. 3 for details)

*α*2	*d*_N_ ± SE	*d*_S_ ± SE	*d*_N_/*d*_S_	*S*_nt_	*S*_aa_	π
Black-tailed godwit						
PBR	0.340 ± 0.113	0.344 ± 0.172	0.988	8	9	0.268
Non-PBR	0.047 ± 0.011	0.093 ± 0.030	0.505	10	22	0.042
Red-billed gull						
PBR	0.236 ± 0.108	0.179 ± 0.118	1.318	5	7	0.191
Non-PBR	0.037 ± 0.010	0.057 ± 0.020	0.649	7	17	0.028
Red knot						
PBR	0.428 ± 0.170	0.271 ± 0.132	1.579	7	7	0.320
Non-PBR	0.072 ± 0.013	0.145 ± 0.032	0.497	19	35	0.055

The godwit MHC-I organization, considering classical and non-classical genes, was more similar to the knots than to the gulls, both knots and godwits for example have putatively classical MHC-I alleles with and without a 3 bp deletion (Table 3). Interestingly, non-classical MHC genes have been reported in both knots and gulls, clearly seen as alleles with low diversity in significantly supported clusters that are distant from other alleles in the phylogenetic neighbour-networks presented below. However, we did not find any evidence of such non-classical genes in the godwit.

**Table 3:** Number of putatively classical and non-classical MHC-I alleles in three species within the order Charadriiformes. Range of loci and number of alleles per individual (given in brackets), within a sample of 84 Icelandic black-tailed godwits (*Limosa limosa islandica*) and eight red knots (*Calidris canutus*). For the red-billed gulls (*Larus scopulinus*), individual information regarding number of alleles was not available and instead information refers to the total number of loci identified across a sample of 470 individuals

Species	Classical alleles				Non-classical alleles
	Total	Short	Long	Minor	
Black-tailed godwit^a^	1–4 (2–7)	0–2 (0–3)	1–3 (1–6)	0	0
Red knot^b^	2–4 (3–8)	0–2 (0–3)	1–3 (2–5)	0	1–2 (2–4)^c^
Red-billed gull^d^	2	0	1	1	2

^a^This study

^b^Buehler et al. (2013)

^c^One of the genes could possibly be a pseudogene

^d^Cloutier et al. (2011)

**Fig. 4:**
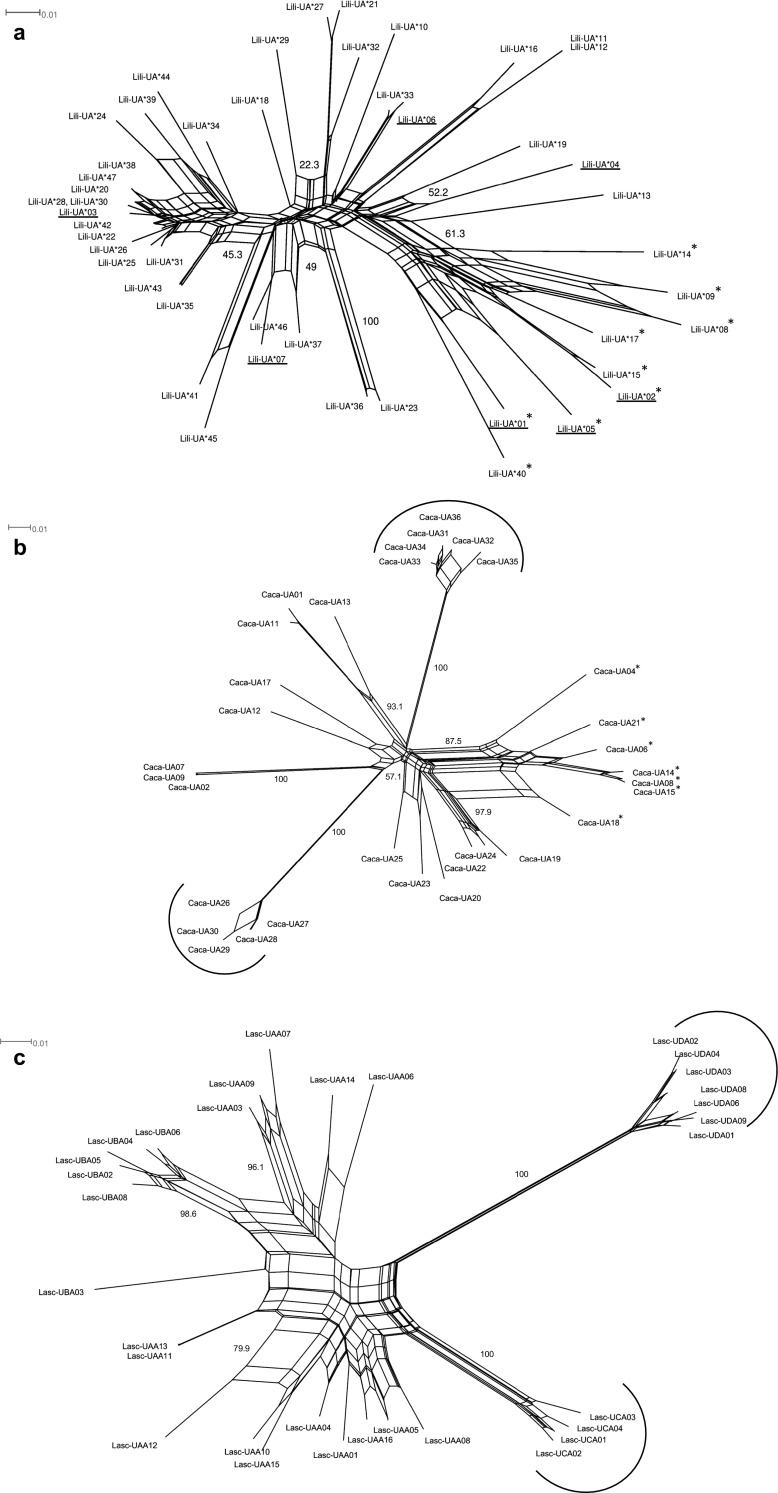
Neighbour-net phylogenetic networks of MHC-I exon 3 nucleotide alleles from **a** Icelandic black-tailed godwits (*Limosa limosa islandica*), **b** red knots (*Calidris canutus* Caca-UA*01-02; Caca-UA*04; Caca-UA*06-09; Caca-UA*11-15; Caca-UA*17-36; Buehler et al. 2013), and **c** red-billed gulls (*Larus scopulinus*, Lasc-UAA*01; Lasc-UAA*03-16; Lasc-UBA*02-06; Lasc-UBA*08; Lasc-UCA*01-04; Lasc-UDA*01-04; and Lasc-UDA*06, 08, and 09; Cloutier et al. 2011). The godwit network is based on 47 alleles, and the alleles that are found with both Sanger and Illumina sequencing are *underlined*. The knot and the gull networks are based on 32 alleles that were downloaded from the GenBank database. Alleles with a 3 bp deletion are indicated by an *asterisk*, and *brackets* indicate clades with alleles that have putatively non-classical or pseudogene function. Bootstrap values after 1000 repeats for main splits are presented

We constructed phylogenetic neighbour-networks built on MHC-I exon 3 alleles for each of the three Charadriiformes species. In the godwit network, the MHC-I alleles formed a single undifferentiated cluster, though the short alleles clustered together, separate from the long alleles (Fig. 4a). However, these short alleles had branches of equal or longer length than the long alleles, indicating that the short alleles are at least as diverse as the long alleles in godwits. Unlike the godwit, the networks of knot and gull MHC-I alleles displayed very high bootstrap support (100 on both) for some distant clusters holding alleles with low diversity, these clusters are thought to contain putatively non-classical alleles (Cloutier et al. 2011; Buehler et al. 2013, Fig. 4b, c). One similarity between the godwit and knot networks is that the short alleles cluster separately with rather high bootstrap support, 61.3 and 87.5 for godwits and knots, respectively.

**Fig. 5:**
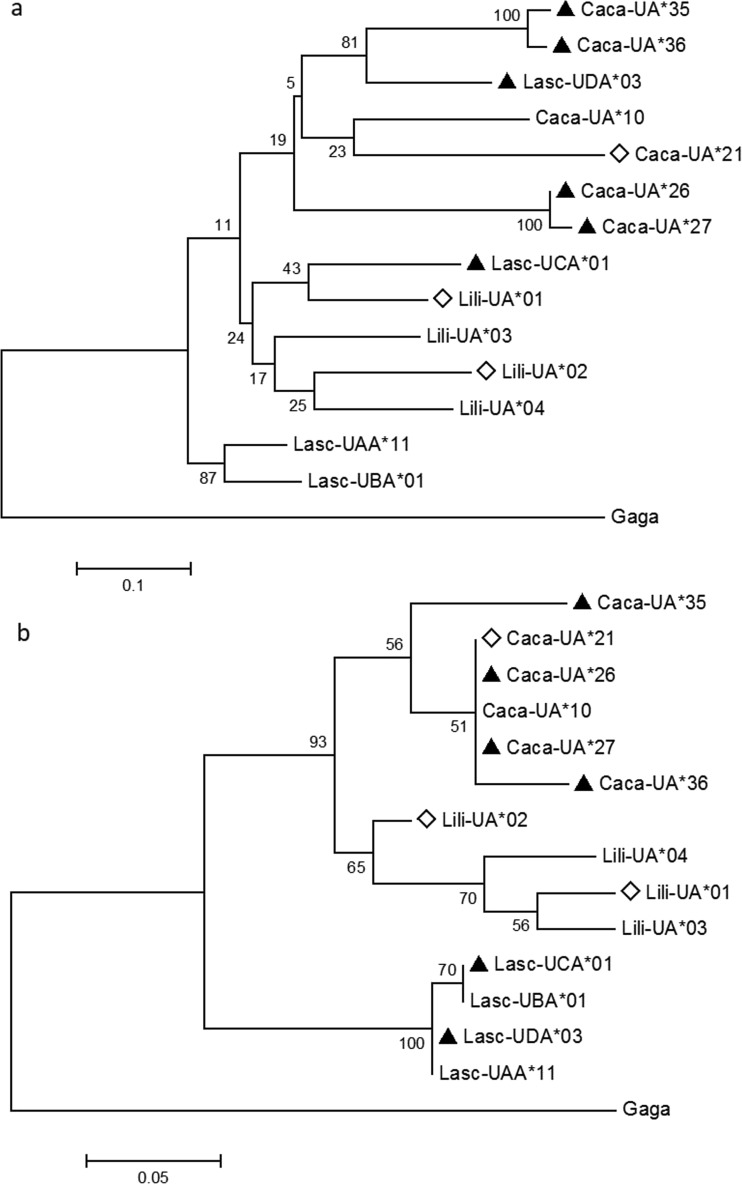
Phylogenetic reconstructions using the maximum likelihood method of MHC-I amino acid sequences from three Charadriiformes species, Icelandic black-tailed godwits (Lili, *Limosa limosa islandica*), red knots (Caca, *Calidris canutus*), and red-billed gulls (Lasc, *Larus scopulinus*), and with domestic chicken (Gaga, *Gallus gallus domesticus*) as outgroup. The trees were built based on **a** exon 2 and 3 or **b** exon 4. Putatively classical alleles (Lasc-UAA*11, Lasc-UBA*01, Caca-UA*10, Caca-UA*21, and Lili-UA*01 to Lili-UA*04) without any deletions, are unmarked in the trees, putatively classical alleles with a 3 bp deletion (i.e., short alleles) are indicated with a *white diamond* (Lili-UA*01, Lili-UA*02, and Caca-UA*21), and non-classical alleles are indicated with a *black triangle* (Lasc-UCA*01, Lasc-UDA*03, Caca-UA*26, Caca-UA*27, Caca-UA*35, and Caca-UA*36 (Cloutier et al. 2011; Buehler et al. 2013)). *Numbers on branches* indicate bootstrap values after 1000 repeats

Phylogenetic reconstructions of amino acid sequences from the three Charadriiformes species, based on exons 2–3 and 4 separately, resulted, as expected, in trees with different features and bootstrap support. The phylogenetic reconstruction of exons 2 and 3 (the regions that contain the PBR) had low bootstrap support in the deeper nodes, and significant support was only found in a few terminal nodes. This tree placed one non-classical gull MHC-I allele among the godwit alleles and another non-classical gull MHC-I allele among non-classical knot alleles (Fig. 5a, nucleotide tree in online resource Fig. S2a). Interestingly, the two 3 bp deletion alleles in godwits were separated in this tree and not in a single cluster, as they were in the network analysis. A gene tree that perfectly matched the species tree appeared when the phylogenetic reconstruction was based on exon 4 amino acid sequences from godwits, gulls, and knots. The bootstrap support was considerably higher in the exon 4 tree compared to the exon 2–3 tree (Fig. 5b; nucleotide tree in online resource Fig. S2b). Exon 4 is subject to purifying selection within species, it encodes the CD8 binding site, and is known to be less variable than exons 2–3 that are subject to balancing selection.

## Discussion

In this study, MHC-I was partly characterized in a long-distance migrant wader, the Icelandic black-tailed godwit. We isolated four long transcripts from a single individual, covering the major part of the extra-cellular MHC-I protein. MHC-I exon 3 was then genotyped successfully in 84 individuals using Illumina MiSeq, and in total, we verified 47 different nucleotide alleles, i.e., exon 3 sequences, with and without a 3 bp deletion. Godwits have between one and seven putatively classical MHC-I alleles in open reading frame per individual, and our preliminary data suggests that at least five alleles are expressed. Previous studies of MHC-I in two other Charadriiformes species, the knot and the gull, have reported the occurrence of both putatively classical and non-classical MHC-I genes. Non-classical MHC alleles were defined by having low diversity and forming significantly supported clusters in phylogenetic networks (Cloutier et al. 2011; Buehler et al. 2013). Using this definition of putatively non-classical MHC-I alleles, we did not detect any non-classical alleles in godwits. In godwits, the short alleles with a 3 bp deletion display a similar or even slightly higher diversity than the long alleles without a 3 bp deletion.

The godwit MHC-I transcripts (733–736 bp) showed typical characteristics of functional antigen-presenting genes (Halenius et al. 2015), e.g., highly conserved peptide main-chain sites, inter- and intra-domain contact residues, and CD8 binding sites (Bjorkman et al. 1987; Saper et al. 1991; Grossberger and Parham 1992; Kaufman et al. 1994). The only exception was the godwit allele Lili-UA*03 where tyrosine (Y) had been replaced by histidine (H) at position 170, a known peptide anchoring site in the peptide main-chain. This substitution is likely to have an impact on the MHC protein, since the amino acids tyrosine (Y) and histidine (H) have very different chemical properties, tyrosine is aromatic and histidine is basic (Betts and Russell 2003). Nevertheless, the same substitution (Y to H) in the same peptide anchoring position has been found in classical MHC genes in humans, mice, and knots and seems to not affect the function (Shum et al. 1999; Buehler et al. 2013), though most likely the binding properties of the MHC protein. When examining this position in 40 additional MHC-I alleles from Illumina MiSeq genotyping, we found four more alleles where tyrosine (Y) was replaced by histidine (H), and also six alleles where tyrosine (Y) was substituted with phenylalanine (F). A tyrosine (Y) replacement by phenylalanine (F), in the same peptide anchoring position, has previously been observed in other birds, mallards, and knots but was then associated with putatively non-classical alleles (Moon et al. 2005; Buehler et al. 2013).

MHC class I alleles in godwits are of two different lengths, one codon is absent in exon 3 in some alleles. The short alleles (with a 3 bp deletion) were detected with both Sanger and Illumina MiSeq sequencing, and they tended to have a more even distribution in the godwit population than long alleles. Most godwit individuals (79%) had both long and short alleles. Alleles with 3 bp deletions have been frequently reported among birds of the order Passeriformes (Alcaide et al. 2013; O’Connor et al. 2016). Within the order Charadriiformes, a 3 bp deletion, likely at the same position as in godwits, was reported in classical MHC-I genes in the phylogenetically closer knot but not in the more distant gull (Cloutier et al. 2011; Buehler et al. 2013). The short alleles have some unique motifs that are not found in the long alleles, e.g., “GENE” at position 148–152, likewise the long alleles have unique motifs that are not found in the short alleles, e.g., “EDGTV” at position 147–151. These two motifs in short and long alleles are found both in godwits and knots (Buehler et al. 2013). Since both types of alleles are found in godwits and knots, but not in gulls, it could indicate that long and short alleles were present in their *Scolopacidae* common ancestor. However, neither the phylogenetic reconstruction on exons 2 and 3, nor a network analysis based on exon 3 sequences from godwits and knots (data not shown), support the short alleles in godwits and knots being orthologous. One explanation for this lack of support could be that the godwit and knot split a rather long time ago (app. 57 million years ago (Ma); Baker et al. 2007) and that the signal of the trans-species phylogenetic relationship among long and short alleles has been lost even though certain motifs still remain.

That PBR are subjected to positive selection is a well-known feature of classical MHC alleles. We found between two and four positively selected sites in godwit MHC-I exon 3 alleles, though some of these sites fell outside the PBR inferred from chickens and humans (Wallny et al. 2006). Knots and gulls have a larger number of residues in exon 3 subjected to positive selection than godwits, seven sites in knots, and 7–12 sites in gulls (Cloutier et al. 2011; Buehler et al. 2013). The limited number of sites subject to positive selection in exon 3 of godwits could be due to limited selection from pathogens. However, it is likely that the high rates of synonymous substitution in the PBR plays a significant role, particularly so for short alleles. High rates of synonymous substitutions have been previously reported for MHC-I alleles among both passerine and non-passerine species (Westerdahl et al. 1999; Alcaide et al. 2013; Gonzalez-Quevedo et al. 2015). As in the studies of positive selection in the PBR of passerines, multiple MHC gene copies were included in the analyses due to the difficulty of locus assignment, and this approach could also make it more difficult to detect sites subject to positive selection.

Comparison of MHC diversity among three species within Charadriiformes showed that godwit MHC-I alleles exhibit intermediate levels of nucleotide diversity and evolutionary divergence compared to knots and gulls. Outside the order Charadriiformes, our data on godwit MHC diversity is similar to those described for other non-passerine species (Strandh et al. 2011; Alcaide et al. 2013). Nonetheless, the godwit MHC diversity is still lower than that commonly reported in passerines (Schut et al. 2011; Sepil et al. 2012; Gonzalez-Quevedo et al. 2015). In contrast to godwits, the high number of alleles per individual in passerines may be associated with their shorter lifespan, rapid evolutionary rate, and larger effective population sizes, factors that allow higher effectiveness of positive selection (Takahata 1990; Welch et al. 2008; Alcaide et al. 2013).

Recombination, gene conversion, and point mutation play important roles in MHC gene evolution (Hess and Edwards 2002; Spurgin et al. 2011). Recombination events in putatively classical MHC genes have been reported in knots and gulls (Buehler et al. 2013; Cloutier et al. 2011) and also in species from the bird orders, Passeriformes, Galliformes, and Falconiformes (Alcaide et al. 2009; Wutzler et al. 2012; Alcaide et al. 2013; Zeng et al. 2016). In the godwits, we only found support for recombination in a small subset of the MHC alleles, i.e., a single recombination point for short alleles. However, recombination events are also indicated by the multiple reticulate and parallel splits seen among godwit alleles in the network topology.

Despite the thorough sequencing of MHC alleles in a single godwit individual with several primer combinations and deep sequencing with Illumina MiSeq in 84 individuals, we saw no evidence for godwits having non-classical MHC genes. Non-classical genes have been reported in both knots and gulls, where both species have at least two non-classical genes (Cloutier et al. 2011; Buehler et al. 2013). These non-classical genes can easily be seen in the neighbour-networks of both knots and gulls, where they form distant clusters with high bootstrap support. In the godwit neighbour-networks, no such distant clusters can be seen. Regarding the number of MHC loci, godwits have one to four loci (2–7 alleles) per individual, whereas knots have up to six loci and gulls up to four loci (Cloutier et al. 2011; Buehler et al. 2013). However, when focusing on only putatively classical loci, and considering the presence of short alleles, the MHC-I organization in godwits is considerably more similar to knots than to gulls. For example, both godwits and knots have 0–3 short alleles per individuals and 1–6 (2–5 for knots) long alleles. This higher similarity between godwit and knot MHC is expected, since the separation between gulls (Lari) and waders (Scolopaci) occurred earlier (around the beginning of late Cretaceous; app. 89 Ma), than the split between *Calidris* spp. and *Limosa* spp. which happened around late Paleocene (app. 57 Ma) (Baker et al. 2007).

It has been proposed that migration may impose a higher selection from pathogens on birds than residency (Westerdahl 2007; Westerdahl et al. 2014). It is interesting to note that godwits and knots, which are both migratory, had slightly higher MHC diversity and divergence in our study than gulls, which are resident. Although this pattern fits the prediction of migratory birds being under higher pressure from pathogens, it is difficult to make any general conclusions from comparing such a small number of species. Even though godwits and knots have similar MHC diversity, the godwits had fewer sites subject to positive selection. One important difference between our study and the knot study by Buehler et al. (2013), which might explain some differences regarding the MHC diversity and number of sites subject to positive selection, is that we investigated only one subspecies, the Icelandic godwit, known to stay in low pathogen areas, whereas Buehler et al. (2013) investigated two subspecies (*Calidris canutus islandica* and *Calidris canutus rufa*) with different migratory strategies and pathogen exposures (Buehler and Piersma 2008). The MHC-I diversity in the study by Buehler et al. (2013) was to a very large extent based on *C. c. rufa*, seven out of eight genotyped individuals were from the *C. c. rufa* population, hence, the knot subspecies that is expected to be exposed to a higher selection pressure from pathogens than the subspecies *C. c. islandica*. So, regarding lifestyle and migratory strategies, the Icelandic godwits investigated in the present study are more similar to *C. c. islandica*, and the low diversity in these godwits could reflect their low exposure to pathogens resulting in a limited selection from pathogens. It would indeed be interesting to investigate the MHC diversity and divergence in a larger number of *C. c. islandica* individuals and compare their diversity with Icelandic godwits, and also to study additional subspecies of godwits, for example, the nominate (*L. l. limosa*), which is likely to be subject to a different selection pressure from pathogens, than the Icelandic subspecies.

## Conclusions

Godwits seem to possess only putatively classical MHC-I genes which contrasts with two other Charadriiformes species, gulls, and knots that have both putatively classical and non-classical genes. Godwit MHC-I alleles have few sites subject to positive selection compared to gulls and knots, and we believe that a potential explanation is a limited selection from pathogens in godwits, particularly so for the Icelandic subspecies of black-tailed godwits that we have investigated in the present study.

## Electronic supplementary material

ESM 1 (PDF 671 kb)
