# Supplementary material for: Characterization of MHC class I in a long distance migratory wader, the Icelandic black-tailed godwit
Source: Immunogenetics. 2017 May 23;69(7):463–78. doi: 10.1007/s00251-017-0993-7 (PMC5486808; doi:10.1007/s00251-017-0993-7)
Supplement: Supplementary file 1 — (PDF 671 kb) [file 251_2017_993_MOESM1_ESM.pdf]

Online resource

Characterization of MHC class I in a long distance migratory wader, the Icelandic black-tailed godwit

Sara Pardal[1*], Anna Drews[2*], José A. Alves[3,4], Jaime A. Ramos[1], Helena Westerdahl[2]

[1]MARE - Marine and Environmental Sciences Centre, Department of Life Sciences, University of Coimbra, 3000-456 Coimbra, Portugal
[2]MEEL - Molecular Ecology and Evolution Lab, Lund University, Ecology building, 223 62 Lund, Sweden
[3]CESAM – Centre for Environmental and Marine Studies, Dep. Biology. University of Aveiro, Campus Universitário de Santiago, 3810-193 Aveiro, Portugal
[4]University of Iceland, South Iceland Research Centre, Fjolheimer, IS-800 Selfoss, Iceland

*Corresponding author: S. Pardal, Marine and Environmental Sciences Centre, Department of Life Sciences, University of Coimbra, 3000-456 Coimbra, Portugal. E-mail: saralpardal@hotmail.com; orcid.org/0000-0003-2594-9450

A. Drews, Molecular Ecology and Evolution Lab, Lund University, Ecology building, 223 62 Lund, Sweden. E-mail: anna.drews@biol.lu.se;

Material and Methods

gDNA and cDNA preparation

Extraction and isolation

Total genomic DNA from the avian blood samples was extracted by an adapted ammonium acetate protocol (Richardson et al. 2001), quantified in NanoDrop and diluted to our working concentration (25 ng/µL). Evaluation of DNA quality was done by amplification of a house-keeping gene like the sex determination CDH1 gene fragment using primers set 2550F (3´-GTTACTGATTCGTCTACGAGA-5´) and 2718R (3´-ATTGAAATGATCCAGTGCTTG-5´) (Fridolfsson and Ellegren 1999). Reaction products were run on 2% agarose gel for band visualization and sex determination of each sample.

RNA extraction and purification was done using a combination of TRIzol LS protocol (Life Technologies, Carlsbad, CA, USA) and RNeasy Mini kit (QIAGEN, Hilden, Germany). In short, an aliquot (200 µl) of blood and RNAlater was transferred to a new tube and centrifuged at 16,000 g for 1 min to remove the RNAlater solution. The sample volume was then adjusted to 250 µl by adding RNAse free water. Homogenization and phase separation was done according to the Trizol LS manufacturer protocol, resulting in an aqueous phase which was transferred to a new tube and one volume of 70% EtOH was added. From this step onwards, extraction was done according to the RNeasy Mini Kit manufacturer protocol including a column DNase treatment. Finally, samples were resuspended in 30 µl of RNase-free water and stored at -80ºC.

Filtering Illumina data

For bioinformatics post-processing of NGS data, we used Amplicon Sequencing Analysis Tools (AmpliSAT) (web server http://evobiolab.biol.amu.edu.pl/amplisat/; Sebastian et al. 2015) for demultiplexing, clustering and filtering. Firstly, and to ensure the quality of the data, sequences with an average Phred quality scores (Q) below 30 were removed with AmpliCLEAN tool. Then the AmpliCHECK tool was used for preliminary overview of de-multiplexed reads and allowed the determination of the clustering and filtering parameters for removing artefacts. The parameters

were set having in mind key assumptions based upon previously described methods; (a) for a reliable allele number characterization, a minimum read depth per amplicon is required (Lighten et al. 2014); (b) sequencing errors such as substitutions and chimeras can occur at high per amplicon frequency (PAF), but always co-occur with the allele(s) from which they were originated (Shao et al. 2013); (c) artefacts generally appear at a lower PAF and in few amplicons (Galan et al. 2010); (d) sequences with incorrect lengths resulting from sequencing errors (insertions/deletions) should be regarded has artefacts (Galan et al. 2010). Once these parameters set, we used the AmpliSAS tool to cluster and filter the data. Reliable read depth was determined based on a linear plot of amplicon read depths; here the required read depth was set to 4000 reads per amplicon. To reassign reads arising from artefacts to the parental sequences from which they arose, the clustering function in AmpliSAS was used. Artefact sequences were only merged with the dominant sequences (presumed parent sequences) if they differed by 1-2 bp and had ≤ 25% of read depth compared to the dominant sequences. However, if a sequence differed by 1-2 bp from the dominant sequences but had more than 25% of read depth, compared to the dominant sequences, it was classified as "subdominants" and formed a new cluster. Next, to remove any remaining artifacts from the dataset, a suitable per amplicon frequency was determined. This was done by finding the best match between technical duplicates (n=6 samples) in a similar fashion to Karlsson and Westerdahl (2013) and O´Connor et al. (2016). Best matches were obtained with a per amplicon frequency of 3.4% and any sequences occurring below this value were considered to be artefacts and removed. Finally, chimeric sequences were identified, and deleted, by using the chimera checking function within AmpliSAS (in our data set the highest frequency of chimeras was 0.6%, hence much lower than the threshold set to 3.4%).

Table S1 - Primers used to cover most of Major Histocompatibility Complex (MHC) class I gene of Icelandic black-tailed godwits, *Limosa limosa islandica*. Star signal "*" stands for primers designed during this study and *T*a for annealing temperature. Partial (p) and full coverage of exons (Ex) were obtained using different primer combinations. The differences in sequence length is because of 3 base pair deletions in some exon 3 sequences

Primer name	Direction	Primer sequence 5´-3´	*T*a (ºC)	Coverage	Sequence length (inner part)	References
*LiliM2F**	Forward	GGCCCCACTCCCTGCGTTAC	65.5	p Ex2 -Ex3 - p Ex4	733 – 736 bp	This study
P126	Reverse	AGTACCRGTGCCGBGTGGAGCA	65.5			Strandh et al. 2011
LiliM2F	Forward	GGCCCCACTCCCTGCGTTAC	65.5	p Ex2 - p Ex3	476 – 479 bp	This study
*A23H8**	Reverse	GAGATACGTGAGCTACGGGC	61.4			
A21P2	Forward	TGGAGGACGGTAGCACCAG	61	p Ex3	174 – 177 bp	Strandh et al. 2011
A23H8	Reverse	GAGATACGTGAGCTACGGGC	61.4			This study
*LiliM23F**	Forward	CAGGGCCCCACTCCCTGC	65.1	p Ex2	229 bp	This study
*LiliM2R**	Reverse	CCGCTACAACCAGAGCVGG	62.4			
LiliM23F	Forward	CAGGGCCCCACTCCCTGC	65.1	Ex2 - Ex3 - p Ex4	741 bp	This study
P126	Reverse	AGTACCRGTGCCGBGTGGAGCA	65.5			Strandh et al. 2011
*LiliM3F**	Forward	TCGYGTTCCAGGGGCTCACA	62.4	p Ex3	244 – 247 bp	This study
*LiliM3R**	Reverse	GGCYGTGCTGGAGAGGAAA	59.9			
*LiliM3F2**	Forward	GGCTGTGASCTCCTGGAGGA	63.5	p Ex3	227 – 230 bp	This study
LiliM3R	Reverse	GGCYGTGCTGGAGAGGAAA	59.9			

Table S2 – Inference of selection on MHC-I exon 3 alleles of Icelandic black-tailed godwits (*Limosa limosa islandica*). The number of codon sites identified to be under selection with SLAC, FEL and REL using the Datamonkey webserver. The analyses were done using three different sets of nucleotide alleles: short and long alleles combined (n = 40 randomly selected nucleotide sequences), for long alleles only (n = 35), or for short alleles (n = 9). We used integrative analysis to combine the results from the three methods. For a site to be considered as selected (positive or negative), the site needed to be subjected to significant selection by at least two maximum likelihood methods, or by one significantly selected and the second with borderline significance values (i.e. close to default *p* values). We call these selected sites, consensus sites

**Number of sites**	**Exon 3 alleles**	**SLAC (P<0.1)**	**FEL (P<0.1)**	**REL (P>50)**	**Consensus sites**
Positively selected	Combined	3	7	6	4
	Long	2	3	14	3
	Short	0	0	13	2
Negatively selected	Combined	9	13	20	11
	Long	9	12	13	8
	Short	1	7	1	2

Table S3 – Confirmed Major Histocompatibility Complex class I (MHC) alleles obtained by cloning and sequencing MHC from one Icelandic black-tailed godwit individual (*Limosa limosa islandica; Lili-UA*). Partial (p) and full coverage of exons 2, 3 and 4 were obtained using different primer combinations. Displayed are the number of clones identified for each allele with the length of the sequences presented in the brackets. When several primer combinations were used, a length distribution is reported

	cDNA				gDNA	
Exon coverage per allele (length/N seq)	p Ex2-3-p Ex4	p Ex2-p Ex3	p Ex2	p Ex3	p Ex3	Total
Lili-UA*01	20 (733 – 738)	5 (476)	4 (229)	24 (174 – 244)	3 (174)	56
Lili-UA*02	12 (733 – 738)	0	0	2 (174)	1 (174)	15
Lili-UA*03	4 (736 – 741)	0	0	2 (247)	0	6
Lili-UA*04	1 (736)	0	0	2 (177)	5 (177)	8
Lili-UA*05	0	1 (476)	0	8 (174 – 244)	3 (174)	12
Lili-UA*06	0	0	0	0	2 (177)	2
Lili-UA*07	0	0	0	0	1 (177)	1
Total number of clones	37	6	4	38	15	**100**

Table S4 – Major histocompatibility class I (Lili-UA) exon 3 alleles. The nucleotide alleles were identified by Illumina MiSeq sequencing of MHC-I from 84 individuals of Icelandic black-tailed godwit (*Limosa limosa islandica*)

# Genotyped individual	Alleles found (gDNA)					
Bird 1	Lili-UA*38	Lili-UA*29	Lili-UA*15	Lili-UA*08		
Bird 2	Lili-UA*47	Lili-UA*38	Lili-UA*20	Lili-UA*14	Lili-UA*09	Lili-UA*04	
Bird 3	Lili-UA*37	Lili-UA*27	Lili-UA*13	Lili-UA*10	Lili-UA*04		
Bird 4	Lili-UA*43	Lili-UA*30	Lili-UA*13	Lili-UA*08	Lili-UA*04		
Bird 5	Lili-UA*40	Lili-UA*38	Lili-UA*23	Lili-UA*09	Lili-UA*07	Lili-UA*04	Lili-UA*03
Bird 6	Lili-UA*40	Lili-UA*34	Lili-UA*28	Lili-UA*17	Lili-UA*05	Lili-UA*04	
Bird 7	Lili-UA*40	Lili-UA*38	Lili-UA*23	Lili-UA*10	Lili-UA*04	Lili-UA*03	
Bird 8	Lili-UA*40	Lili-UA*38	Lili-UA*31	Lili-UA*05	Lili-UA*04	Lili-UA*03	
Bird 9	Lili-UA*29	Lili-UA*20	Lili-UA*18	Lili-UA*15	Lili-UA*07		
Bird 10	Lili-UA*31	Lili-UA*26	Lili-UA*24	Lili-UA*15	Lili-UA*10		
Bird 11	Lili-UA*38	Lili-UA*35	Lili-UA*20	Lili-UA*08			
Bird 12	Lili-UA*28	Lili-UA*15	Lili-UA*08	Lili-UA*07	Lili-UA*04		
Bird 13	Lili-UA*40	Lili-UA*33	Lili-UA*27	Lili-UA*07	Lili-UA*04	Lili-UA*03	
Bird 14	Lili-UA*47	Lili-UA*40	Lili-UA*34	Lili-UA*20	Lili-UA*05	Lili-UA*04	
Bird 15	Lili-UA*34	Lili-UA*15	Lili-UA*04				
Bird 16	Lili-UA*31	Lili-UA*28	Lili-UA*23	Lili-UA*16	Lili-UA*09		
Bird 17	Lili-UA*37	Lili-UA*28	Lili-UA*19	Lili-UA*15	Lili-UA*07	Lili-UA*04	
Bird 18	Lili-UA*40	Lili-UA*38	Lili-UA*34	Lili-UA*18	Lili-UA*05	Lili-UA*04	
Bird 19	Lili-UA*40	Lili-UA*31	Lili-UA*08	Lili-UA*06	Lili-UA*03		
Bird 20	Lili-UA*22	Lili-UA*18	Lili-UA*10	Lili-UA*06	Lili-UA*04		
Bird 21	Lili-UA*38	Lili-UA*15	Lili-UA*08				
Bird 22	Lili-UA*46	Lili-UA*32	Lili-UA*29	Lili-UA*15	Lili-UA*12	Lili-UA*04	
Bird 23	Lili-UA*39	Lili-UA*28	Lili-UA*14	Lili-UA*10	Lili-UA*07	Lili-UA*04	
Bird 24	Lili-UA*29	Lili-UA*12	Lili-UA*11	Lili-UA*04			
Bird 25	Lili-UA*38	Lili-UA*21	Lili-UA*10	Lili-UA*04			
Bird 26	Lili-UA*40	Lili-UA*38	Lili-UA*21	Lili-UA*08	Lili-UA*07	Lili-UA*03	
Bird 27	Lili-UA*38	Lili-UA*29	Lili-UA*23	Lili-UA*15	Lili-UA*14		
Bird 28	Lili-UA*45	Lili-UA*42	Lili-UA*36	Lili-UA*13	Lili-UA*05	Lili-UA*04	Lili-UA*03
Bird 29	Lili-UA*31	Lili-UA*27	Lili-UA*15	Lili-UA*07	Lili-UA*02	Lili-UA*01	
Bird 30	Lili-UA*10	Lili-UA*09	Lili-UA*06				
Bird 31	Lili-UA*40	Lili-UA*20	Lili-UA*10	Lili-UA*07	Lili-UA*06	Lili-UA*03	
Bird 32	Lili-UA*40	Lili-UA*38	Lili-UA*21	Lili-UA*15	Lili-UA*05		
Bird 33	Lili-UA*37	Lili-UA*34	Lili-UA*28	Lili-UA*15	Lili-UA*13	Lili-UA*04	
Bird 34	Lili-UA*38	Lili-UA*15	Lili-UA*10				
Bird 35	Lili-UA*15	Lili-UA*10	Lili-UA*07	Lili-UA*04			
Bird 36	Lili-UA*40	Lili-UA*35	Lili-UA*25	Lili-UA*24	Lili-UA*20	Lili-UA*11	Lili-UA*03
Bird 37	Lili-UA*31	Lili-UA*20	Lili-UA*15	Lili-UA*02			
Bird 38	Lili-UA*31	Lili-UA*19	Lili-UA*12				
Bird 39	Lili-UA*31	Lili-UA*28	Lili-UA*13	Lili-UA*10			
Bird 40	Lili-UA*38	Lili-UA*29	Lili-UA*27	Lili-UA*10	Lili-UA*09	Lili-UA*04	
Bird 41	Lili-UA*44	Lili-UA*28	Lili-UA*21	Lili-UA*19	Lili-UA*10		
Bird 42	Lili-UA*27	Lili-UA*22	Lili-UA*18	Lili-UA*16	Lili-UA*04		

Bird 43	Lili-UA*40	Lili-UA*38	Lili-UA*24	Lili-UA*13	Lili-UA*05	Lili-UA*04	
Bird 44	Lili-UA*32	Lili-UA*31	Lili-UA*29	Lili-UA*28	Lili-UA*12	Lili-UA*10	
Bird 45	Lili-UA*38	Lili-UA*31	Lili-UA*10	Lili-UA*05			
Bird 46	Lili-UA*38	Lili-UA*24	Lili-UA*23	Lili-UA*15	Lili-UA*10		
Bird 47	Lili-UA*40	Lili-UA*38	Lili-UA*09	Lili-UA*03			
Bird 48	Lili-UA*31	Lili-UA*38	Lili-UA*04	Lili-UA*17	Lili-UA*15		
Bird 49	Lili-UA*19	Lili-UA*10					
Bird 50	Lili-UA*23	Lili-UA*19	Lili-UA*12	Lili-UA*29			
Bird 51	Lili-UA*19	Lili-UA*15	Lili-UA*28	Lili-UA*31	Lili-UA*41		
Bird 52	Lili-UA*13	Lili-UA*27	Lili-UA*10	Lili-UA*31	Lili-UA*07		
Bird 53	Lili-UA*15	Lili-UA*09	Lili-UA*38				
Bird 54	Lili-UA*10	Lili-UA*05	Lili-UA*27	Lili-UA*37	Lili-UA*39		
Bird 55	Lili-UA*28	Lili-UA*09	Lili-UA*31	Lili-UA*23	Lili-UA*16		
Bird 56	Lili-UA*10	Lili-UA*09	Lili-UA*38	Lili-UA*23			
Bird 57	Lili-UA*06	Lili-UA*05	Lili-UA*04	Lili-UA*03	Lili-UA*02	Lili-UA*07	
Bird 58	Lili-UA*10	Lili-UA*05	Lili-UA*04	Lili-UA*07	Lili-UA*40	Lili-UA*21	Lili-UA*34
Bird 59	Lili-UA*10	Lili-UA*27	Lili-UA*38	Lili-UA*11			
Bird 60	Lili-UA*15	Lili-UA*28	Lili-UA*07	Lili-UA*06	Lili-UA*17		
Bird 61	Lili-UA*27	Lili-UA*03	Lili-UA*38	Lili-UA*40	Lili-UA*11		
Bird 62	Lili-UA*27	Lili-UA*02	Lili-UA*07	Lili-UA*24	Lili-UA*25		
Bird 63	Lili-UA*08	Lili-UA*20	Lili-UA*38	Lili-UA*35			
Bird 64	Lili-UA*03	Lili-UA*08	Lili-UA*38	Lili-UA*07	Lili-UA*40	Lili-UA*21	
Bird 65	Lili-UA*15	Lili-UA*27	Lili-UA*02	Lili-UA*31	Lili-UA*07	Lili-UA*01	
Bird 66	Lili-UA*10	Lili-UA*04	Lili-UA*27	Lili-UA*07	Lili-UA*17		
Bird 67	Lili-UA*04	Lili-UA*12	Lili-UA*38	Lili-UA*02	Lili-UA*07		
Bird 68	Lili-UA*04	Lili-UA*03	Lili-UA*38	Lili-UA*40	Lili-UA*16		
Bird 69	Lili-UA*04	Lili-UA*09	Lili-UA*13	Lili-UA*38			
Bird 70	Lili-UA*15	Lili-UA*04	Lili-UA*12	Lili-UA*38			
Bird 71	Lili-UA*05	Lili-UA*13	Lili-UA*07	Lili-UA*06			
Bird 72	Lili-UA*05	Lili-UA*04	Lili-UA*07	Lili-UA*40	Lili-UA*25	Lili-UA*33	
Bird 73	Lili-UA*10	Lili-UA*09	Lili-UA*38	Lili-UA*24			
Bird 74	Lili-UA*15	Lili-UA*03	Lili-UA*40	Lili-UA*01	Lili-UA*24	Lili-UA*23	Lili-UA*25
Bird 75	Lili-UA*12	Lili-UA*27	Lili-UA*13	Lili-UA*07	Lili-UA*29		
Bird 76	Lili-UA*15	Lili-UA*08	Lili-UA*28	Lili-UA*38	Lili-UA*37		
Bird 77	Lili-UA*10	Lili-UA*04	Lili-UA*12	Lili-UA*29			
Bird 78	Lili-UA*10	Lili-UA*38	Lili-UA*14				
Bird 79	Lili-UA*15	Lili-UA*04	Lili-UA*08	Lili-UA*38			
Bird 80	Lili-UA*10	Lili-UA*04	Lili-UA*27	Lili-UA*11			
Bird 81	Lili-UA*03	Lili-UA*28	Lili-UA*38	Lili-UA*40	Lili-UA*41		
Bird 82	Lili-UA*10	Lili-UA*38	Lili-UA*23	Lili-UA*16	Lili-UA*32		
Bird 83	Lili-UA*04	Lili-UA*38	Lili-UA*02	Lili-UA*18			
Bird 84	Lili-UA*10	Lili-UA*27	Lili-UA*08	Lili-UA*38	Lili-UA*07		

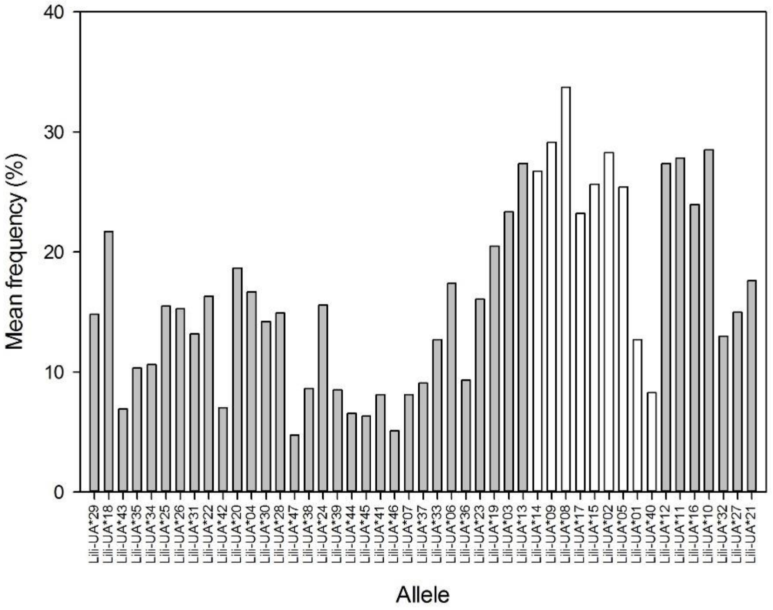

Fig. S1 – Frequency distribution of MHC-I exon 3 alleles from the 84 Icelandic black-tailed godwits (*Limosa limosa islandica*) individuals screened with Illumina MiSeq sequencing. Grey columns indicate long alleles (247 bp), while white columns indicate short alleles (244 bp)

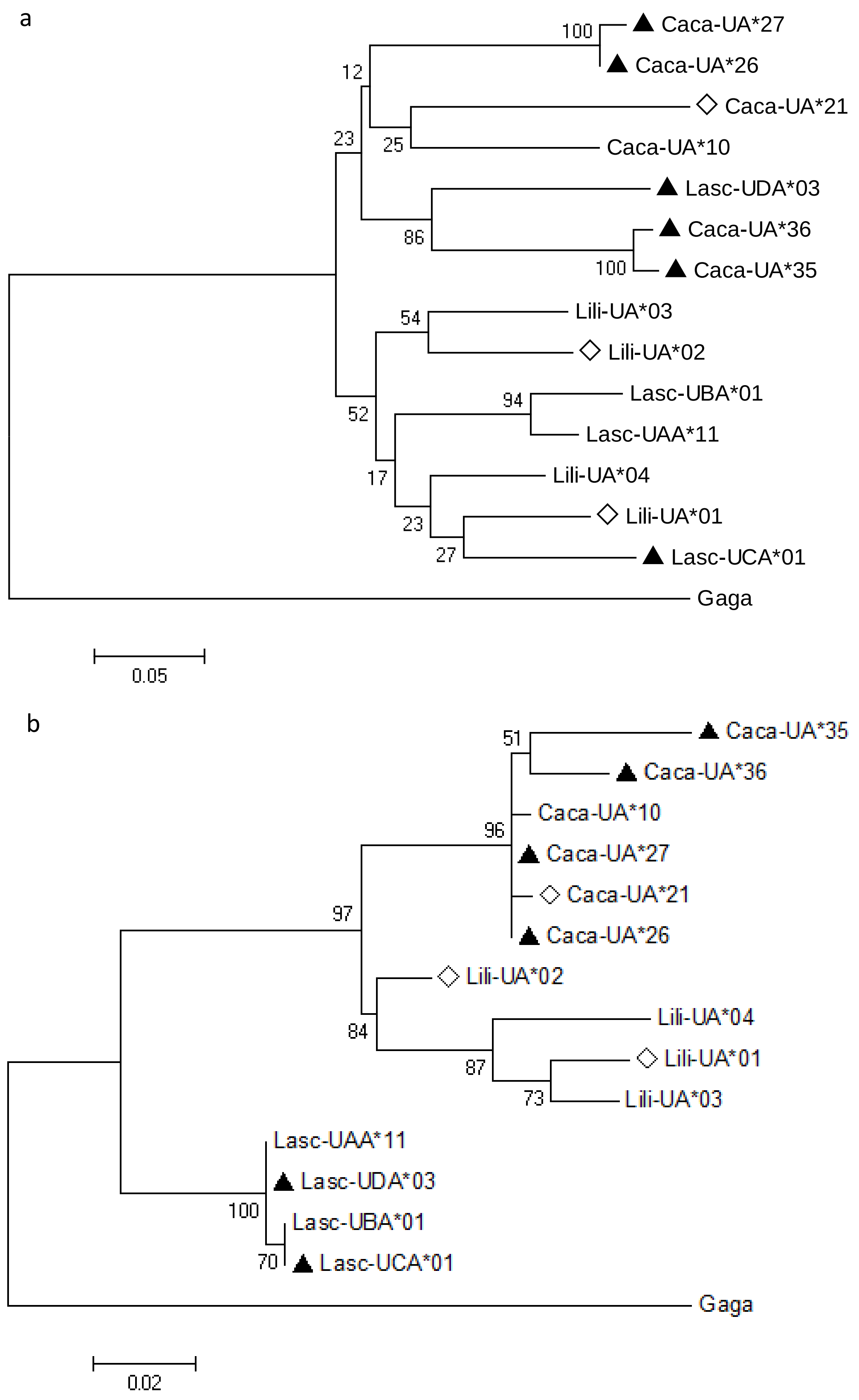

Fig. S2 – Phylogenetic reconstructions using the maximum likelihood method of MHC-I nucleotide sequences from three Charadriiformes species, Icelandic black-tailed godwits (Lili, *Limosa limosa islandica*), red knots (Caca, *Calidris canutus*), and red-billed gulls (Lasc, *Larus*

scopulinus), and with domestic chicken (Gaga, *Gallus gallus domesticus*) as outgroup. The trees were built based on **a** exon 2 and 3 or **b** exon 4. Putatively classical alleles (Lasc-UAA*11, Lasc-UBA*01, Caca-UA*10, Caca-UA*21, and Lili-UA*01 to Lili-UA*04) without any deletions, are unmarked in the trees, putatively classical alleles (short alleles) with a 3 bp deletion are indicated with a *white diamond* (Lili-UA*01, Lili-UA*02, and Caca-UA*21), and non-classical alleles are indicated with a *black triangle* (Lasc-UCA*01, Lasc-UDA*03, Caca-UA*26, Caca-UA*27, Caca-UA*35, and Caca-UA*36 (Cloutier et al. 2011; Buehler et al. 2013)). *Numbers on branches* indicate bootstrap values after 1000 repeats

References

Fridolfsson A-K, Ellegren H (1999) A simple and universal method for molecular sexing of non-ratite birds. Oikos 30:116–121.

Galan M, Guivier E, Caraux G, Charbonnel N, Cosson JF (2010) A 454 multiplex sequencing method for rapid and reliable genotyping of highly polymorphic genes in large-scale studies. BMC Genomics 11:296. doi: 10.1186/1471-2164-11-296

Karlsson M, Westerdahl H (2013) Characteristics of MHC class I genes in house sparrows *Passer domesticus* as revealed by long cDNA transcripts and amplicon sequencing. J Mol Evol 77:8–21. doi: 10.1007/s00239-013-9575-y

Lighten J, van Oosterhout C, Paterson IG, McMullan M, Bentzen P (2014) Ultra-deep Illumina sequencing accurately identifies MHC class IIb alleles and provides evidence for copy number variation in the guppy (*Poecilia reticulata*). Mol Ecol Resour 14:753–767. doi: 10.1111/1755-0998.12225

O’Connor EA, Strandh M, Hasselquist D, Nilsson JÅ, Westerdahl H (2016) The evolution of highly variable immunity genes across a passerine bird radiation. Mol Ecol 25:977–989. doi: 10.1111/mec.13530

Richardson DS, Jury FL, Blaakmeer K, Komdeur J, Burke T (2001) Parentage assignment and extra-group paternity in a cooperative breeder: the Seychelles warbler (*Acrocephalus sechellensis*). Mol Ecol 10:2263–2273. doi: 10.1046/j.0962-1083.2001.01355.x

Sebastian A, Herdegen M, Migalska M, Radwan J (2015) Amplisas: a web server for multilocus genotyping using next-generation amplicon sequencing data. Mol Ecol Resour 16:498–510. doi: 10.1111/1755-0998.12453

Shao W, Boltz VF, Spindler JE, Kearney MF, Maldarelli F, Mellors JW, Stewart C, Volfovsky N, Levitsky A, Stephens RM, Coffin JM (2013) Analysis of 454 sequencing error rate, error sources, and artifact recombination for detection of low-frequency drug resistance mutations in HIV-1 DNA. Retrovirology 10:18. doi: 10.1186/1742-4690-10-18

Strandh M, Lannefors M, Bonadonna F, Westerdahl H (2011) Characterization of MHC class I and II genes in a subantarctic seabird, the blue petrel, *Halobaena caerulea* (Procellariiformes). Immunogenetics 63:653–66. doi: 10.1007/s00251-011-0534-8
